# Japanese experience of newborn screening for lysosomal storage diseases and adrenoleukodystrophy

**DOI:** 10.1186/s13023-025-03848-4

**Published:** 2025-07-24

**Authors:** Takanori Onuki, Makiko Tajika, Yohei Sugiyama, Masaru Shimura, Keiko Ichimoto, Toju Tanaka, Hiromi Nyuzuki, Motomichi Kosuga, Ohsuke Migita, Tetsuya Ito, Hideo Sasai, Ryosuke Bo, Junpei Hamada, Takashi Hamazaki, Norio Sakai, Takahito Inoue, Kimitoshi Nakamura, Torayuki Okuyama, Kei Murayama

**Affiliations:** 1 Department of Metabolism, Center for Medical Genetics, Chiba Children’s Hospital, Chiba, Japan; 2 Division of Pediatrics, Department of Homeostatic Regulation and Development, Graduate School of Medical and Dental Sciences, Niigata University, Niigata, Japan; 3 Department of Pediatrics, National Hospital Organization Hokkaido Medical Center, Sapporo, Japan; 4 Division of Medical Genetics, National Center for Child Health and Development, , Setagaya, Japan; 5 Department of Pediatrics, St. Marianna University School of Medicine, Kawasaki, Japan; 6 Department of Pediatrics, School of Medicine, Fujita Health University, Toyoake, Japan; 7 Department of Pediatrics, Gifu University Graduate School of Medicine, Gifu, Japan; 8 Department of Pediatrics, Kobe University Graduate School of Medicine, Kobe, Japan; 9 Department of Pediatrics, Graduate School of Medicine, Ehime University, Toon, Japan; 10 Department of Pediatrics, Graduate School of Medicine, Osaka Metropolitan University, Osaka, Japan; 11 Center for Promoting Treatment of Intractable Diseases, ISEIKAI International General Hospital, Osaka, Japan; 12 Department of Pediatrics, Fukuoka University Chikushi Hospital, Chikushino, Japan; 13 Department of Pediatrics, Faculty of Life Sciences, Kumamoto University, Kumamoto, Japan; 14 Department of Clinical Genomics, Saitama Medical University, Saitama, Japan; 15 Department of Pediatrics, Juntendo University Faculty of Medicine, Bunkyo City, Japan; 16 Diagnostics and Therapeutic of Intractable Diseases, Intractable Disease Research Center, Graduate School of Medicine, Juntendo University, Hongo 2-1-1, Bunkyo-Ku, Tokyo, 113-8421 Japan

**Keywords:** Newborn screening, NBS, Lysosomal storage disease, LSD, Adrenoleukodystrophy, ALD

## Abstract

**Background:**

Recently, Newborn screening (NBS) has been expanded worldwide to include lysosomal storage diseases (LSDs) and adrenoleukodystrophy (ALD) due to the importance of early diagnosis and early treatment. In Japan, NBS for LSDs, termed expanded NBS, was first implemented in Kumamoto prefecture in 2006 as pilot study. NBS for ALD was subsequently introduced in Aichi prefecture and Gifu prefecture in 2021. Expanded NBS for LSDs and ALD has become more widespread in Japan. In light of this current situation, we considered it is necessary to clarify the usefulness of expanded NBS, prevalence of each disease, challenges encountered. Therefore, we reported the current implementation status of expanded NBS in Japan.

**Method:**

A survey was conducted among physicians responsible for expanded NBS in each target region Japan. The target regions were those that implemented NBS for LSDs and/or ALD for more than one year. The survey items included: the entity conducting expanded NBS, the facilities conducting the tests, the target areas, medical institutions for close examination such as detailed biochemical analysis and/or genetic sequencing, and treatments, types of target diseases, fee for NBS, sample collection methods, testing method, and quantitative data on expanded NBS, retesting, and diagnoses in each area.

**Results:**

Responses were received from nine regions and an organization (CReARID). The total number of 733,838 newborns were screening, with 101 diagnoses: 75 cases of Fabry disease, 10 of mucopolysaccharidosis (MPS) II, 8 of Pompe disease, 5 of Gaucher disease, 2 of MPS I, 1 of ALD, respectively) were diagnosed. More cases were diagnosed with the target disease than the estimated prevalence. In contrast, the positive predictive value was low and false-positive rates was elevated, particularly for PD, MPS II, and ALD, have been attributed to pseudodeficiency alleles and methodological differences. Moreover, variant of unknown significance (VUS) in the *ABCD1* gene was detected in many of the patients with suspected ALD.

**Conclusion:**

In Japan, Expanded NBS for LSDs and ALD has become more widespread. Since its implementation, some patients have been diagnosed and received treatment. However, challenges such as pseudodeficiency, indications, testing methods, and VUS that require improvement.

## Background

Lysosomal storage diseases (LSDs) are caused by the intracellular accumulation of substrates that are normally degraded owing to the deficiency or reduced activity of lysosomal enzymes. Enzyme replacement therapy, substrate reduction therapy, pharmacological chaperone therapy, and hematopoietic stem cell transplantation, have been developed and implemented in clinical practice [1]. Adrenoleukodystrophy (ALD) is an X-linked peroxisomal disorder and caused by pathogenic variants in the *ABCD1* gene [2]. The most severe form of ALD, the pediatric cerebral type, is a progressive disease that begins at mean 7.1 years of age and is characterized by behavioral abnormalities, vision and hearing loss, gait disturbance, and neurodevelopmental regression [2]. The observation that early hematopoietic stem cell transplantation is associated with significant improvements in survival and functional outcomes has been reported [2]. In addition, emerging ex vivo gene therapies, such as lentiviral hematopoietic stem cell gene therapy for ALD and MLD, have also shown effectiveness [2–5]. The discussion on including these conditions in newborn screening (NBS) remains highly active for these reasons. Because pre-onset diagnosis of LSDs and ALD is both challenging and crucial to the availability of specific treatments, NBS for these diseases is expected to be beneficial. Consequently, NBS for several treatable LSDs and ALD has been implemented in several countries [4–6].

In Japan, Tajima et al. created a modified recommended uniform screening panel (RUSP) score based on the original RUSP [7, 8]. According to the modified RUSP score, the list includes spinal muscular atrophy, primary immunodeficiency disease, ALD, and LSDs such as pompe disease (PD), mucopolysaccharidosis (MPS) I and II, and others.

NBS for LSDs, termed expanded NBS, was first implemented in Kumamoto prefecture in 2006 as pilot study [9, 10]. For ALD started in Aichi prefecture and Gifu prefecture in 2021 [11]. Expanded NBS has expanded from small-scale studies conducted by individual researchers to other regions of the country in the form of self-funded studies. In light of this current situation, we considered it is necessary to clarify the usefulness of expanded NBS, prevalence of each disease, challenges encountered. Therefore, we reported the current implementation status of expanded NBS in Japan.

## Material and methods

A questionnaire survey on the expanded NBS was conducted in Japan between December 2023 and April 2024. The target regions were those that had been conducting NBS for LSDs and/or ALD for over one year. All targeted regions responded to the questionnaire survey. None of them were invalid and excluded.

### Subjects and target regions

**Fig. 1:**
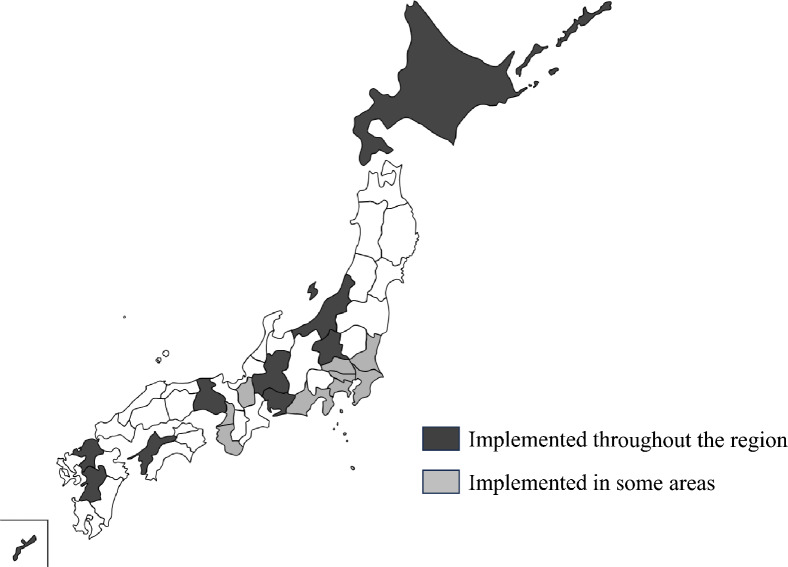
Expanded NBS Area Map in Japan. A map showing areas where expanded NBS for LSDs/ALD is implemented in Japan. Black areas represented areas where NBS is implemented throughout the regions: Hokkaido prefecture, Niigata prefecture, Gunma prefecture, Gifu prefecture, Aichi prefecture, Hyogo prefecture, Ehime prefecture, Fukuoka prefecture, Okinawa prefecture. The grey areas are areas that are implemented in some regions; Ibaraki prefecture, Saitama prefecture, Chiba prefecture, Tokyo Metropolis, Kanagawa prefecture, Shizuoka prefecture, Shiga prefecture, Osaka prefecture, and Wakayama prefecture

The target regions were Hokkaido prefecture, Niigata prefecture, Chiba prefecture, Tokyo Metropolis, Kanagawa prefecture, Aichi prefecture, Gifu prefecture, Osaka prefecture, Osaka City, Hyogo prefecture, Ehime prefecture, Fukuoka prefecture, Kumamoto prefecture and an organization (Clinical and Research Association for Rare, Intractable Diseases: CReARID) that conducts expanded NBS in some regions. There region and CReARLD were surveyed (Fig. 1). Questionnaires were sent to representative physicians of each target region.

### Survey

The survey items included: the entity conducting expanded NBS, the facilities conducting the tests, the target areas, medical institution for a close examination such as detailed biochemical analysis and/or genetic sequencing, and treatments, types of target diseases, fee for NBS (whether public funds were used), sample collection methods ( if dried blood spot (DBS) was used for both conventional and expanded NBS or if separate samples were newly collected for expanded NBS), testing method, and quantitative data on expanded NBS, retesting, and diagnoses in each area.

## Results

Responses were received from nine regions and one organization (CReARID), comprising ten responses that formed the dataset of this study.

### The entity conducting expanded NBS, the facilities conducting the tests, and the target areas

The results are presented in Table 1 and Fig. 1. The CReARID conducts NBS in 12 regions, including Chiba prefecture, Tokyo Metropolis, Saitama prefecture, Gunma prefecture. Approximately half of the six regions tested both entities and conducted expanded NBS at the same facility.

**Table 1:** Main organizations, target area, number of affiliated hospitals

	Main organizations	Target area	Number of hospitals
1	Hokkaido network for early diagnosis of rare diseases *	Hokkaido pref	More than 4
2	Clinical & Research Association for Rare, Intractable Diseases(CReARID) *	Hokkaido pref.^‡^ Tokyo Metlopolis^‡^、 Gunma pref.、Saitama pref.^‡^ Chiba pref.^‡^ Ibaraki pref.^‡^ Kanagawa pref.^‡^ Shizuoka pref.^‡^ Wakayama pref.^‡^ Shiga pref.^‡^ Okinawa pref	1 ~ 5
3	The Association of Children’s Rare Diseases in Niigata(ASCRN) *	Niigata pref	2
4	Aichi Rare Disease neteork(ARDnet) *	Aichi pref	2
5	Tokai Masscreening Promotion Association(TOMAS) *	Gifu pref	1
6	Osaka City Environmental Health Association **	Osaka city	4
7	Hyogo Children`s Advanced Medical Council*	Hyogo pref	5
8	Ehime Children`s Advanced Medical Council*	Ehime pref	1
9	IBUKI*	Fukuoka pref	4
10	Japan Council for Advanced Pediatric Therapy*	Kumamoto pref	1

*General Incorporated Association **General Incorporated Foundation

‡ Limited to some areas

pref., prefecture

### Medical institution numbers

The medical institutions responsible for detailed biochemical analysis and/or genetic sequencing and treatments are listed in Table 1. Some prefectures had only one institution for examination of NBS-positive cases, whereas others had multiple institutions (two—five) for examination. Genetic sequencing and/or detailed biochemical analysis including measurement of accumulated substances (such as blood Lyso-GB3, blood Lyso-GB1, and urinary glucosaminoglycans [GAGs]) and enzyme activity were performed at each medical institution. For FD, the maternal family history was evaluated for symptoms suggestive of FD, such as renal failure, urinary mulberry bodies, hypertrophic cardiomyopathy, hypoacusis, stroke and acute neuropathic pain. In addition, genetic sequencing and/or detailed biochemical analysis of blood Lyso-GB1 was performed with their consent. *GLA* genetic sequencing was checked whether the variant corresponded to a previously reported classical type. The diagnosis was confirmed through the integration of diagnostic test, laboratory analyses, clinical evaluation. In addition, unconfirmed cases such as female FD carriers were followed-up after undergoing ethical review.

### DBS for expanded NBS

In five areas, expanded NBS was performed using DBS from the same filter paper as that used for conventional NBS, and in the remaining six areas, new ones were collected. In Aichi, Gifu, and Osaka prefectures, two filter papers were collected, even though the facilities conducting the tests were the same for both conventional and expanded NBS. In contrast, in Niigata and Hyogo prefectures, the same filter paper was used for both conventional and expanded NBS, even though they were tested at different facilities.

### Types of target diseases

The types of disease screened for are shown in Table 2. PD, MPS I, MPS II and FD screening was performed across all regions. FD screening was performed only in boys in the CReARID organization, as well as in Aichi and Niigata prefectures. MPS IVA and VI screeing were performed in the CReARID and Osaka City, while MPS VII screening was limited to only Osaka City. ALD screening was performed only in boys in the CReARID and Aichi and Gifu prefectures.

**Table 2:** Summary of the target diseases by region

	PD	MPSI	MPSII	MPSIVA	MPSVI	MPSVII	GD	FD	ALD
1.Hokkaido pref	●	●	●				●	●	
2.CReARID	●	●	●	●	●			●*	●*
3.Niigata pref	●	●	●					●*	
4.Aichi pref	●	●	●					●*	●*
5.Gifu pref	●	●	●				●	●	●*
6.Osaka City	●	●	●	●	●	●	●	●	
7.Hyogo pref	●	●	●				●	●	
8.Ehime pref	●	●	●				●	●	
9.Fukuoka pref	●	●	●				●	●	
10.Kumamoto pref	●	●	●				●	●	

●Target disease, *screening performed in boys only, pref., prefecture

### Testing method

Most of the regions screened for LSDs using liquid chromatograph Tandem mass spectrometry (LC–MS/MS) or MS/MS, while the rest performed screening via digital microfluidics fluorimetry using the 4-Methylumbelliferone (4-MU) (Table 3). In Hokkaido prefecture, initial testing was performed using 4-MU, and second-tier testing employed LC–MS/MS or MS/MS. NBS for ALD was performed by measuring C26:0 lysophosphatidylcholine (C26:0-LPC) and C24:0-LPC levels using high-performance liquid chromatography-MS/MS.

**Table 3:** Implementation status in each region and institution

	Method	Total number	Observed incidence
Hokkaido pref	First:4MU 2nd:LC–MS/MS, MS/MS	38,981	MPS II 2(1: 19,500)、FD 3(1: 13,000)
CReARID	LC–MS/MS, MS/MS	89,568	PD 2(1:48,000) 、MPS II 4(1:45,000) FD(1:5,500)、ALD 1(1:34,000)
Niigata pref	LC–MS/MS, MS/MS	13,785	FD 2(1:6,900)
Aichi pref	LC–MS/MS, MS/MS	212,644	PD 5(1:42,00)、MPS I 1(1:160,000)、 MPS II 3(1:53,000) 、FD 27(1:6,000)
Gifu pref	4-MU	25,380	FD 4(1:6,250)
Osaka City	LC–MS/MS, MS/MS	7,958	FD 1(1:8,000)
Hyogo pref	LC–MS/MS, MS/MS	18,945	MPS I 1(1:19,000)、FD 1(1:19,000)
Ehime pref	4-MU	15,345	N/A
Fukuoka pref	4-MU	154,567	PD 1(1:275,000)、MPS II 1(1:155,000)、 GD 1(1:155,000)、FD 17(1:9,100)
Kumamoto pref	4-MU	156,665	GD 4(1:14,100)、FD 11(1:23,000)
Total		733,838	101(1: 7,200)

pref., prefecture

### NBS Fee

The fee for expanded NBS was covered by the patient in 55.6% (5/9 region). Public funding eligibility was restricted, with full subsides available in some areas and partial subsidies for screening in others.

### Expanded NBS results by region

Tables 3 and 4 show the expanded NBS results by region. The total number of examinees was 733,838. The newborns that were re-examined ranged from 1/230 (0.43%) to 1/4200 (0.024%) and varied among the regions. The number of newborns requiring genetic sequencing and/or detailed biochemical analysis also varied among the regions (1/650–1/6,500). A total of 101 newborns were diagnosed, with FD being the most common, followed by MPS II and PD.

**Table 4:** Summary of expanded NBS data by region

	Total number of examinees	Number of patient re-examined	Number of patients requiring genetic sequencing and/or detailed biochemical analysis	Observed incidence	positive predictive value
PD	861,379	678(1:1,000)	398(1: 2,100)	8(1: 100,000)	2.0%
MPS I	628,212	493(1:1,200)	84(1: 7,500)	2(1: 310,000)	2.4%
MPS II	621,176	408(1:1,500)	312(1: 2,000)	10(1: 62,000)	3.2%
GD	329,769	97(1:3,400)	48(1: 6,800)	5(1: 65,000)	10.4%
FD	803,366	807(1:1,000)	240(1: 3,400)	75(1: 10,000)	31.3%
ALD	138,038	79(1:2,000)	86(1: 1,600)	1(1: 140,000)	1.2%

The ratios of re-examination, genetic sequencing and/or detailed biochemical analysis, and diagnosis are shown in parentheses. The number of diagnoses varied by region; however, the total incidence rates were generally higher than those previously reported. However, many patients who underwent genetic sequencing and/or detailed biochemical analysis showed false-positive results. Therefore, detailed examination: incidence rate ratios were high

#### PD

3.7.2. Approximately 1 in 2,000 newborns underwent a genetic sequencing and/or detailed biochemical analysis for PD and eight patients were diagnosed. The frequency of diagnosis was approximately 1 in 100,000, which was less frequent than the frequency of PD previously diagnosed using NBS (1/34,400) and higher than the general incidence of 1/91,000 in Japan [12, 13]. Among the newborns requiring genetic sequencing and/or detailed biochemical analysis, positive predictive value (PPV) was 2% (1/50), and false-positive rate was high.

#### MPS I

3.7.4. Approximately one in 7,500 newborns underwent genetic sequencing and/or detailed biochemical analysis, and two were diagnosed. The frequency of diagnosis is approximately one in 310,000, which is higher than the general incidence of 1/1,110,000 in Japan [13]. PPV was 2.4%.

#### MPS II

3.7.6. Approximately 1 in 2,000 newborns underwent genetic sequencing and/or detailed biochemical analysis, and 10 were diagnosed. The frequency of diagnosis was approximately 1 in 62,000, which were higher frequent than the frequency of MPS II previously diagnosed using NBS (1/197,700) and the general incidence of 1/263,000 in Japan [13, 14]. Among the newborns requiring genetic sequencing and/or detailed biochemical analysis, PPV was 3.3%.

#### GD

3.7.8. Approximately 1 in 7,000 newborns underwent genetic sequencing and/or detailed biochemical analysis, and five were diagnosed. The frequency of diagnosis was approximately 1 in 65,000, which is higher than the frequency of GD previously diagnosed using NBS (1/77,720) and the general incidence of 1 in 526,000 in Japan [13, 15]. PPV was 10.4%, the diagnosis rate was particularly high in Kumamoto prefecture.

#### FD

Approximately 1 in 3,400 newborns underwent genetic sequencing and/or detailed biochemical analysis, and 75 were diagnosed. The frequency of diagnosis was approximately 1 in 10,000, which is approximately the same as the frequency of FD previously diagnosed using NBS (1/7,057) and higher than the general incidence (1/80,000) in Japan [13, 16]. PPV was 31.3%, which was relatively high for NBS for LSDs.

#### ALD

Approximately 1 in 1,600 newborns underwent genetic sequencing and/or detailed biochemical analysis, and one was diagnosed. The frequency of diagnosis was approximately 1 in 140,000, which is higher than the general incidence (1/500,000) in Japan [13]. PPV was 1.2%. Additionally, variants of unknown significance (VUS) were detected in 16 newborns.

## Discussion

Our report provides the first comprehensive summary of expanded NBS implementation in Japan. Over the implementation period spanning of up to 11 years (average of six years), more than 730,000 newborns were tested. 101 newborns were diagnosed with target diseases: 75 with FD, 10 with MPS II, 8 with PD, 5 with GD, 2 with MPS I, 1 with ALD. These findings demonstrate that expanded NBS is gradually gaining traction across Japan. Although our report focused on regions and organizations that had conducted expanded NBS for at least one year, the program has since been implemented in additional regions, and this trend is expected to continue expanding in Japan.

In the present study, more cases were diagnosed with the target disease than the estimated prevalence [12–16]. This may be due to the wide range the disease severities, especially among LSDs. This is expected to result in many undiagnosed patients. The targeted LSDs and ALD are rare diseases that pose challenges for timely diagnosis. For example, FD, the most frequently diagnosed condition in our study, is often identified late in current clinical practice. A delay in diagnosis leads to late initiation of therapy when organ damage has already occurred, by which time disease-specific treatments may have limited benefit compared to early treatment [17]. Given that early diagnosis and treatment correlate with improved patient outcomes, NBS is considered a useful means to resolve these challenges.

There are some reports that included and discussed NBS for LSDs and from other countries [4–6, 18, 19]. For LSDs, nearly all countries and regions screen by measuring enzyme activities using DBS [6, 18–21], although some parts of Italy also measured accumulated substances such as Lyso-Gb1, Lyso-Gb3 and GAGs as second-tier tests [18]. The prevalence of each country varied because of genetic disorders, PPV was also not so high. However, in Italy PPV was increased dramatically by measuring accumulated substances [18]. On the other hands, in ALD, all countries and regions employed C26:0-LPC measurement via DBS [5]. In addition, several countries and regions have included *ABCD1* gene sequencing as third or fourth tier [5]. A significant increase in registered VUS in the *ABCD1* variant database has been noted since the implementation of NBS [5]. These reports from other countries have shown similar results to our study and suggest that improvements are needed.

Several challenges have been noted with expanded NBSs. First, the false-positive rate, especially for PD, MPS II and ALD, was significantly high. Moreover, PPV of PD, MPS I, MPS II, and ALD were 2%, 2.4%, 3.2%, and 1.2%, respectively (Table 3). We believe that one of the reasons for this is pseudodeficiency, which is LSD specific. The pseudodeficiency allele (p.G576S) in *GAA* gene is prevalent in individuals of Asian descent, including the Japanese population [22]. In addition, in the Asian population, the high frequency of pseudodeficiency alleles in *IDS* gene gives rise to a high false-positive rate [23], highlighting need for second-tier test in the screening system. Proposed solutions include optimizing of cut-off values, the measurement of accumulated substances other than enzyme activity, and testing method. In fact, Kumamoto and Fukuoka Prefectures (who performed NBS using 4-MU) and CReARID (who performed using LC–MS/MS), which adjusted the cut-off values, reduced the rate of precision examinations (Table 5). However, adjusting cut-off alone is not a complete solution. Pseudodeficiency does not increase accumulated substrate levels which can be clarified through biochemical assays. For example, GAGs that accumulate in MPS can be quantified using various methods such as enzyme-linked immunosorbent assay and LC–MS/MS using DBS [24, 25]. Simultaneous measurement of enzyme activity could distinguish patients with MPS from healthy control or pseudodeficiency [18, 26, 27]. Moreover, secondary GAG testing offer key advantages over molecular testing, including lower testing costs, reduced turnaround time, and avoidance of identifying cases with uncertain molecular results [28]. In PD, the creatine/creatinine to GAA ratio was reported to be useful to improve the specificity [20]. Combining these approaches could help address these issues. Regarding analytical methodology, differences in observed incidence and false-positive rates between MS/MS or LC–MS/MS and 4-MU testing methods were also observed (Table 6). The analytical precision of the MS/MS method is higher than that for digital microfluidics fluorimetry [29]. On the other hands, comparisons between MS/MS or LC–MS/MS and 4-MU do not indicate the superior performance of one method over the other [21]. Our study showed that the MS/MS or LC–MS/MS method had a lower false-positive rate and higher PPV than the 4-MU method, especially for FD, PD and MPS II (Table 6). In addition, based on differences in the observed incidence, undiagnosed cases may exist in 4-MU method. Such problems not only force unnecessary tests on newborns who would not need them but also lead to a psychological burden on their parents.

**Table 5:** The number of cases requiring detailed examination due to revision of the cut-off value

Target disease	Implementation period	Number of examinees	Number of screening positive (rate)	Observed incidence(positive predictive value)	False positive rate
Fukuoka pref. and Kumamoto pref. (Method:4-MU)					
PD	2013 ~ 2016	112,993	113 (0.1%)	0 (0%)	100%
PD	2017 ~ 2023	318,908	92 (0.03%)	1 (1.1%)	98.9%
CReARID (Method:LC–MS/MS)					
MPS II	2019 ~ 2020	22,935	18 (0.08%)	1 (5.6%)	94.4%
MPS II	2021 ~ 2023	66,469	15 (0.02%)	3 (20%)	80%

In areas where the two tests differed, the screening positive rate and false positive rate were reduced by revising the cut-off value

**Table 6:** Differences in false-positive rates and positive predictive value by test method

	MS/MS、LC–MS/MS				4-MU			
Target disease	Number of examinees	genetic sequencing and/or detailed biochemical analysis	Observed incidence (positive predictive value)	false positive rate	Number of examinees	genetic sequencing and/or detailed biochemical analysis	Observed incidence (positive predictive value)	false positive rate
PD	388,753	172	7 (4.1%)	96%	472,626	226	1 (0.44%)	99.6%
MPS I	337,460	68	2 (3.0%)	97%	290,752	7	0 (0%)	100%
MPS II	330,424	120	9 (8.0%)	92%	290,752	192	1 (0.52%)	99.5%
GD	57,926	43	0 (0%)	100%	271,849	5	5 (100%)	0%
FD	284,525	70	43 (61%)	39%	472,626	144	32 (22%)	78%

MS/MS and LC–MS/MS methods had a lower-false positive rates and higher-positive rate than the 4-MU method except gaucher disease

A second challenge involves managing patients with mild symptoms. According to Wilson and Jungner’s criteria [30], diseases for which NBS is indicated are serious health problems, presenting with early symptoms, and cost-effectiveness is important. Diseases that develop late, such as late-onset FD and late-onset PD, may not be recommended for screening during the neonatal period. Moreover, detecting cases with uncertain clinical significance, such as female FD carriers, raises ethical concerns. However, initiating treatment after the disease has developed and symptoms have progressed may be less effective, and screening for diseases that may develop later is an important issue for the future. It is also important to note that genealogical diagnosis allows for pre-onset diagnosis in the parents and relatives of the originating child.

The third challenge is the detection of VUS. In particular, most of patients who have been required genetic sequencing and/or detailed biochemical analysis for ALD showed VUS of *ABCD1* gene. Because expanded NBS detected elevated plasma levels of very long chain fatty acids, a diagnosis was not made, but followed up was required. In addition, *ABCD1* mutations are known to have poor genotype–phenotype correlations [31]. Therefore, even if NBS diagnosed the disease before the onset of symptoms, the prognosis is difficult to predict.

The establishment of testing methods and cut-off values that ensure a low false-positive rate and maintain true-positive cases, and the establishment of a follow-up system for positive and false-positive cases remain key challenges for the future.

This study has several limitations. First, long-term follow up of screened population were not available. False-negative results cannot be ruled out. Moreover, our survey did not investigate disease severity, and thus the clinical phenotype of confirmed cases remain unclear. This also includes diseases with limited benefit from early diagnosis and treatment, such as Gaucher disease type 2. Second, we did not conduct a cost-effectiveness analysis of expanded NBS. Cost-effectiveness is an important factor in screening tests and should be considered in the future. It is essential to address these issues continuously to refine implementation strategies.

## Conclusion

In Japan, Expanded NBS for LSDs and ALD has become more widespread. Patients have been diagnosed and received treatment. However, challenges such as pseudodeficiency, indications, testing methods, and VUS detection require improvement. These issues necessitate continuous refinement of quantification methods for accumulated substances, cut-off values, and methodologies to optimize screening accuracy while minimizing unnecessary interventions.

## Data Availability

The datasets used and/or analysed during the current study are available from the corresponding author on reasonable request.

## References

[1] Martina JA, Raben N, Puertollano R. SnapShot: lysosomal storage diseases. Cell. 2020;180(3):602-602.e1. 10.1016/j.cell.2020.01.017. 32032518 10.1016/j.cell.2020.01.017 PMC8411567

[2] Gupta AO, Raymond G, Pierpont EI, Kemp S, McIvor RS, Rayannavar A, Miller B, Lund TC, Orchard PJ. Treatment of cerebral adrenoleukodystrophy: allogeneic transplantation and lentiviral gene therapy. Expert Opin Biol Ther. 2022;22(9):1151–62. 10.1080/14712598.2022.2124857. 36107226 10.1080/14712598.2022.2124857

[3] Biffi A, Montini E, Lorioli L, et al. Lentiviral hematopoietic stem cell gene therapy benefits metachromatic leukodystrophy. Science. 2013;341(6148):1233158. 10.1126/science.1233158. 23845948 10.1126/science.1233158

[4] Videbæk C, Melgaard L, Lund AM, Grønborg SW. Newborn screening for adrenoleukodystrophy: International experiences and challenges. Mol Genet Metab. 2023;140(4): 107734. 10.1016/j.ymgme.2023.107734. 37979237 10.1016/j.ymgme.2023.107734

[5] Zhu J, Eichler F, Biffi A, Duncan CN, Williams DA, Majzoub JA. The changing face of adrenoleukodystrophy. Endocr Rev. 2020;41(4):577–93. 10.1210/endrev/bnaa013. 32364223 10.1210/endrev/bnaa013 PMC7286618

[6] Chang S, Zhan X, Liu Y, et al. Newborn screening for 6 lysosomal storage disorders in China. JAMA Netw Open. 2024;7(5): e2410754. 10.1001/jamanetworkopen.2024.10754. 38739391 10.1001/jamanetworkopen.2024.10754 PMC11091758

[7] American College of Medical Genetics Newborn Screening Expert Group. Newborn screening: toward a uniform screening panel and system--executive summary. Pediatrics. 2006; 117(5 Pt 2):S296–307. 10.1542/peds.2005-2633I. 10.1542/peds.2005-2633I 16735256

[8] Tajima Go, Koremura K. Expansion of target diseases for neonatal screening in Japan and in foreign countries. J Jpn Pediatr Soc. 2022;126(1):25–34 (**(in Japanese)**).

[9] Sawada T, Kido J, Yoshida S, Sugawara K, Momosaki K, Inoue T, Tajima G, Sawada H, Mastumoto S, Endo F, Hirose S, Nakamura K. Newborn screening for Fabry disease in the western region of Japan. Mol Genet Metab Rep. 2020;11(22): 100562. 10.1016/j.ymgmr.2019.100562. 10.1016/j.ymgmr.2019.100562 PMC6961758 31956509

[10] Sawada T, Kido J, Sugawara K, Momosaki K, Yoshida S, Kojima-Ishii K, Inoue T, Matsumoto S, Endo F, Ohga S, Hirose S, Nakamura K. Current status of newborn screening for Pompe disease in Japan. Orphanet J Rare Dis. 2021;16(1):516. 10.1186/s13023-021-02146-z. 34922579 10.1186/s13023-021-02146-z PMC8684119

[11] Shimozawa N, Takashima S, Kawai H, Kubota K, Sasai H, Orii K, Ogawa M, Ohnishi H. Advanced diagnostic system and introduction of newborn screening of adrenoleukodystrophy and peroxisomal disorders in Japan. Int J Neonatal Screen. 2021;7(3):58. 10.3390/ijns7030058. 34449525 10.3390/ijns7030058 PMC8395936

[12] Momosaki K, Kido J, Yoshida S, Sugawara K, Miyamoto T, Inoue T, Okumiya T, Matsumoto S, Endo F, Hirose S, Nakamura K. Newborn screening for Pompe disease in Japan: report and literature review of mutations in the GAA gene in Japanese and Asian patients. J Hum Genet. 2019;64(8):741–55. 10.1038/s10038-019-0603-7. 31076647 10.1038/s10038-019-0603-7

[13] Koto Y, Sakai N, Lee Y, Kakee N, Matsuda J, Tsuboi K, Shimozawa N, Okuyama T, Nakamura K, Narita A, Kobayashi H, Uehara R, Nakamura Y, Kato K, Eto Y. Prevalence of patients with lysosomal storage disorders and peroxisomal disorders: A nationwide survey in Japan. Mol Genet Metab. 2021;133(3):277–88. 10.1016/j.ymgme.2021.05.004. 34090759 10.1016/j.ymgme.2021.05.004

[14] Hattori Y, Sawada T, Kido J, Sugawara K, Yoshida S, Matsumoto S, Inoue T, Hirose S, Nakamura K. Frequency of iduronate-2-sulfatase gene variants detected in newborn screening for mucopolysaccharidosis type II in Japan. Mol Genet Metab Rep. 2023;28(37): 101003. 10.1016/j.ymgmr.2023.101003. 10.1016/j.ymgmr.2023.101003 PMC10694771 38053932

[15] Sawada T, Kido J, Sugawara K, Yoshida S, Matsumoto S, Shimazu T, Matsushita Y, Inoue T, Hirose S, Endo F, Nakamura K. Newborn screening for Gaucher disease in Japan. Mol Genet Metab Rep. 2022;18(31): 100850. 10.1016/j.ymgmr.2022.100850. 10.1016/j.ymgmr.2022.100850 PMC8866142 35242582

[16] Inoue T, Hattori K, Ihara K, Ishii A, Nakamura K, Hirose S. Newborn screening for Fabry disease in Japan: prevalence and genotypes of Fabry disease in a pilot study. J Hum Genet. 2013;58(8):548–52. 10.1038/jhg.2013.48. 23677059 10.1038/jhg.2013.48

[17] Germain DP, Altarescu G, Barriales-Villa R, Mignani R, Pawlaczyk K, Pieruzzi F, Terryn W, Vujkovac B, Ortiz A. An expert consensus on practical clinical recommendations and guidance for patients with classic Fabry disease. Mol Genet Metab. 2022;137(1–2):49–61. 10.1016/j.ymgme.2022.07.010. 35926321 10.1016/j.ymgme.2022.07.010

[18] Gragnaniello V, Cazzorla C, Gueraldi D, Puma A, Loro C, Porcù E, Stornaiuolo M, Miglioranza P, Salviati L, Burlina AP, Burlina AB. Light and shadows in newborn screening for lysosomal storage disorders: eight years of experience in Northeast Italy. Int J Neonatal Screen. 2023;10(1):3. 10.3390/ijns10010003. 38248631 10.3390/ijns10010003 PMC10801488

[19] Kubaski F, Sousa I, Amorim T, Pereira D, Silva C, Chaves V, Brusius-Facchin AC, Netto ABO, Soares J, Vairo F, Poletto E, Trometer J, Souza A, Ranieri E, Polo G, Hong X, Herbst ZM, Burlina A, Gelb MH, Giugliani R. Pilot study of newborn screening for six lysosomal diseases in Brazil. Mol Genet Metab. 2023;140(1–2):107654. 10.1016/j.ymgme.2023.107654. 37507255 10.1016/j.ymgme.2023.107654 PMC12516792

[20] Tortorelli S, Eckerman JS, Orsini JJ, Stevens C, Hart J, Hall PL, Alexander JJ, Gavrilov D, Oglesbee D, Raymond K, Matern D, Rinaldo P. Moonlighting newborn screening markers: the incidental discovery of a second-tier test for Pompe disease. Genet Med. 2018;20(8):840–6. 10.1038/gim.2017.190. 29095812 10.1038/gim.2017.190

[21] Millington DS, Bali DS. Current state of the art of newborn screening for lysosomal storage disorders. Int J Neonatal Screen. 2018;4(3):24. 10.3390/ijns4030024. 33072946 10.3390/ijns4030024 PMC7548896

[22] Chien YH, Hwu WL, Lee NC. Newborn screening: Taiwanese experience. Ann Transl Med. 2019;7(13):281. 10.21037/atm.2019.05.47. 31392193 10.21037/atm.2019.05.47 PMC6642927

[23] Chan MJ, Liao HC, Gelb MH, et al. Taiwan national newborn screening program by tandem mass spectrometry for mucopolysaccharidoses Types I, II, and VI. J Pediatr. 2019;205:176–82. 10.1016/j.jpeds.2018.09.063. 30409495 10.1016/j.jpeds.2018.09.063 PMC6623979

[24] Khan SA, Mason RW, Kobayashi H, Yamaguchi S, Tomatsu S. Advances in glycosaminoglycan detection. Mol Genet Metab. 2020;130(2):101–9. 10.1016/j.ymgme.2020.03.004. 32247585 10.1016/j.ymgme.2020.03.004 PMC7198342

[25] de Ruijter J, de Ru MH, Wagemans T, Ijlst L, Lund AM, Orchard PJ, Schaefer GB, Wijburg FA, van Vlies N. Heparan sulfate and dermatan sulfate derived disaccharides are sensitive markers for newborn screening for mucopolysaccharidoses types I. II and III Mol Genet Metab. 2012;107(4):705–10. 10.1016/j.ymgme.2012.09.024. 23084433 10.1016/j.ymgme.2012.09.024

[26] Arunkumar N, Vu DC, Khan S, Kobayashi H, Ngoc Can TB, Oguni T, Watanabe J, Tanaka M, Yamaguchi S, Taketani T, et al. Diagnosis of mucopolysaccharidoses and mucolipidosis by assaying multiplex enzymes and glycosaminoglycans. Diagnostics. 2021;11:1347. 10.3390/diagnostics11081347. 34441282 10.3390/diagnostics11081347 PMC8394749

[27] Ayodele O, Fertek D, Evuarherhe O, Siffel C, Audi J, Yee KS, Burton BK. A systematic literature review on the global status of newborn screening for mucopolysaccharidosis II. Int J Neonatal Screen. 2024;10(4):71. 10.3390/ijns10040071. 39449359 10.3390/ijns10040071 PMC11503380

[28] Li JW, Mao SJ, Chao YQ, Hu CX, Qian YJ, Dai YL, Huang K, Shen Z, Zou CC. Application of tandem mass spectrometry in the screening and diagnosis of mucopolysaccharidoses. Orphanet J Rare Dis. 2024;19(1):179. 10.1186/s13023-024-03195-w. 38685110 10.1186/s13023-024-03195-w PMC11059687

[29] Gelb MH, Lukacs Z, Ranieri E, Schielen PCJI. Newborn screening for lysosomal storage disorders: methodologies for measurement of enzymatic activities in dried blood spots. Int J Neonatal Screen. 2019;5(1):1. 10.3390/ijns5010001. 30957052 10.3390/ijns5010001 PMC6448570

[30] Wilson JM, Jungner YG. Principles and practice of mass screening for disease. Bol Oficina Sanit Panam. 1968;65(4):281–393. 4234760

[31] Kemper AR, Brosco J, Comeau AM, Green NS, Grosse SD, Jones E, Kwon JM, Lam WK, Ojodu J, Prosser LA, Tanksley S. Newborn screening for X-linked adrenoleukodystrophy: evidence summary and advisory committee recommendation. Genet Med. 2017;19(1):121–6. 10.1038/gim.2016.68. 27337030 10.1038/gim.2016.68 PMC5182180

